# PiezoMEMS Nonlinear Low Acceleration Energy Harvester with an Embedded Permanent Magnet †

**DOI:** 10.3390/mi11050500

**Published:** 2020-05-15

**Authors:** Nathan Jackson

**Affiliations:** Center for High Technology Materials & Mechanical Engineering Department, University of New Mexico, Albuquerque, NM 87106, USA; njack@unm.edu; Tel.: +1-505-272-7095

**Keywords:** energy harvester, MEMS, bandwidth

## Abstract

Increasing the power density and bandwidth are two major challenges associated with microelectromechanical systems (MEMS)-based vibration energy harvesting devices. Devices implementing magnetic forces have been used to create nonlinear vibration structures and have demonstrated limited success at widening the bandwidth. However, monolithic integration of a magnetic proof mass and optimizing the magnet configuration have been challenging tasks to date. This paper investigates three different magnetic configurations and their effects on bandwidth and power generation using attractive and repulsive magnetic forces. A piezoMEMS device was developed to harvest vibration energy, while monolithically integrating a thick embedded permanent magnet (NdFeB) film. The results demonstrated that repulsive forces increased the bandwidth for in-plane and out-of-plane magnetic configurations from <1 to >7 Hz bandwidths. In addition, by using attractive forces between the magnets, the power density increased while decreasing the bandwidth. Combining these forces into a single device resulted in increased power and increased bandwidth. The devices created in this paper focused on low acceleration values (<0.1 g) and low-frequency applications.

## 1. Introduction

Vibration-based energy harvesting systems have been extensively investigated over the past decade as a method to create self-sustaining systems [1]. In particular, microelectromechanical systems (MEMS)-based energy harvesters have attracted interest due to their size and potential ability to power wireless sensor networks for the Internet of Things (IoT) [2,3]. Energy harvesting transducers aim to convert one form of energy into usable electrical energy. Vibration energy harvesters convert mechanical energy to electrical energy through electrostatic, electromagnetic, triboelectric, or piezoelectric channels. MEMS-based vibrational energy harvesting devices have two major limitations that have limited their success, namely their (i) low power density and (ii) narrow bandwidth. MEMS devices often have high Q-factors, which helps in increasing power, but at the cost of narrowing the bandwidth. Typical linear MEMS energy harvesters have narrow bandwidths of 1–2 Hz and operate at low resonant frequency (<250 Hz) [4,5]. Most applications have wider frequency spectrums that can change due to various external conditions or time, and if the frequency change is greater than the bandwidth of the energy harvester then the power generated is significantly reduced. In addition, most applications have low acceleration (<0.1 g), but the majority of energy harvesting research focuses on higher acceleration of up to 1 g. Off-resonant frequency devices that operate at high acceleration have demonstrated some success for specific applications, such as tire pressure monitor systems and pacemakers [6,7].

Numerous methods have been investigated to increase the bandwidth of MEMS vibration energy harvesters, including nonlinear structures [8,9,10,11], mechanical impact stoppers [12,13], multiple vibration modes [14], sliding masses via solids [15,16,17] or liquids [18,19,20,21], and various other methods [22]. One method that has been investigated extensively is the development of bistable energy harvesters [23]. One particular method of creating a nonlinear structure involves coupling a magnetic proof mass with stationary magnetic structures [24,25,26]. The magnetic force does not require any additional energy input and functions by effectively altering the stiffness in order to increase the bandwidth [27]. However, these methods have used macroscale permanent magnets. To date, this technology has not been used with an embedded permanent magnet material. In addition, these methods focus on widening the bandwidth using nonlinear dynamics; this typically results in significantly lowering the power density, which is not desired. Previous reports have investigated both in-plane magnetic configurations [28,29], where the magnets are placed along the same horizontal axis, and out-of-plane magnetic configurations [25], where the magnets are placed along the vertical axis (axis of cantilever displacement). Typically, these devices are based on repulsive magnetic forces consisting of S–S or N–N magnetic polarity, so that as the magnets get closer together the magnetic repulsion force increases, creating nonlinear dynamic displacement.

This paper investigates the power and bandwidth effects of varying magnetic nonlinear energy harvesters with varying magnetic configurations. These effects are tested on a piezoelectric MEMS device with embedded permanent magnets. This is the first time this technology has been demonstrated using monolithically integrated magnets. Embedded permanent magnets typically involve using a polymeric binder such as polymethyl methacrylate, polyvinylidene fluoride, or an elastomer. However, these suffer from low fill factors, which significantly decrease the remanence value. This paper uses a recently developed method of increasing the fill factor by eliminating the polymeric binder with a chemical vapor deposited polymer capping layer [30]. Other techniques that could be used in future applications include 3D printed permanent magnetic structures [31]. The magnets used in this paper were embedded in silicon structures, which were either part of the cantilever or were diced and bonded onto the cantilever as stationary magnets. The magnetic strength of these embedded magnets was significantly lower than commercial bulk magnets but was able to microfabricate the magnets brings numerous advantages, such as custom shapes. This paper is an extended version of one that proceeded at the PowerMEMS conference [32], which investigates more magnetic configurations, has extended analysis, and provides extensive characterization.

The aim of the study is to determine the effects of varying the polarity of the magnets to create repulsive and attractive forces in order to alter both bandwidth and power. The devices developed in this study were designed to operate at low frequency (<250 Hz) and low acceleration (<0.1 g). The effects of repulsive magnetic forces have been investigated at higher acceleration, but this study aims to investigate these effects at lower acceleration values (<0.15 g). Research to date has focused on using repulsive forces to widen the bandwidth, however this paper investigates using repulsive, attractive, and combinations of the two forces in order to demonstrate that it is possible to increase the bandwidth without significantly decreasing the power. This paper demonstrates that by combining altering forces in various magnetic configurations, a customizable device can be developed for different applications, depending on power and bandwidth specifications.

## 2. Materials and Methods

### 2.1. Concept

**Figure 1:**
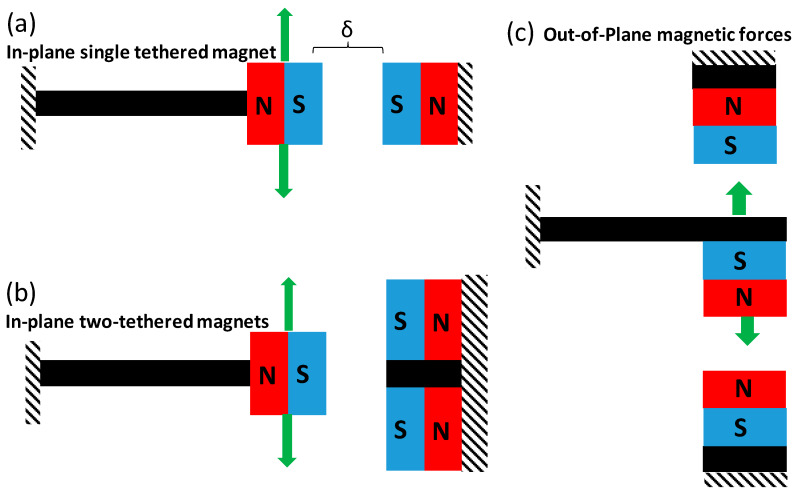
Schematic of magnetic configurations on a cantilever beam. Arrows represent direction of motion of cantilever beam. (**a**) In-plane single magnet configuration with δ (distance) between magnets, which can have attractive or repulsive forces, (**b**) In-plane two-tethered magnetic configuration with repulsive forces and (**c**) out-of-plane magnetic forces with tethered magnets, which can be configured with repulsive or attractive forces.

Three different magnetic configurations were investigated in this study, as shown in Figure 1. These include a single in-plane configuration, as shown in Figure 1a, which can have either an attractive or repulsive force, depending on the polarity of the magnets. The second configuration was an in-plane configuration with two tethered magnets, as shown in Figure 1b. In this setup, there were magnets on the top and bottom of a stationary silicon structure. The magnetic forces on the stationary structure can either be (a) both repulsive, (b) both attractive, or (c) one repulsive and one attractive with respect to the movable magnet by varying the pole locations. The magnet spacing between the stationary structure and movable cantilever were controlled using a micromanipulator that was clamped to the stationary structure. The magnets were developed by embedding a neodymium iron boron (NdFeB) powder with a total thickness of 425 μm into a silicon cavity. The fabrication of the magnets is described in more detail below which followed a similar procedure as previously described [30]. The third configuration that was investigated was an out-of-plane configuration that consisted of a movable cantilever and two fixed devices with magnetic proof mass. This configuration could also include repulsive or attractive forces and combinations of the two, depending on the polarity of the magnets.

The concept of the magnetic devices was based on generating magnetic forces to couple with magnetic proof mass on the cantilever in order to produce nonlinear dynamic displacements. In the case of the out-of-plane configuration, the magnets increase their repelling force as the cantilever displacement amplitude increases thus causing a nonlinear effect. In the case of the in-plane configuration, the magnetic field or repulsion force was largest when the cantilever beam was at its original position and decreased when displacement was large. An attractive S–N magnetic force will have the opposite effect and increase the cantilever amplitude [33]. For example, an increase in displacement of an out-of-plane configuration will increase the magnetic force, which will further increase displacement and stress or strain, thus increasing the power generated. The attractive magnets effectively reduce the stiffness of the cantilever beam. Therefore, as repulsive forces can increase bandwidth and decrease power, attractive forces should increase power.

### 2.2. Microfabrication

**Figure 2:**
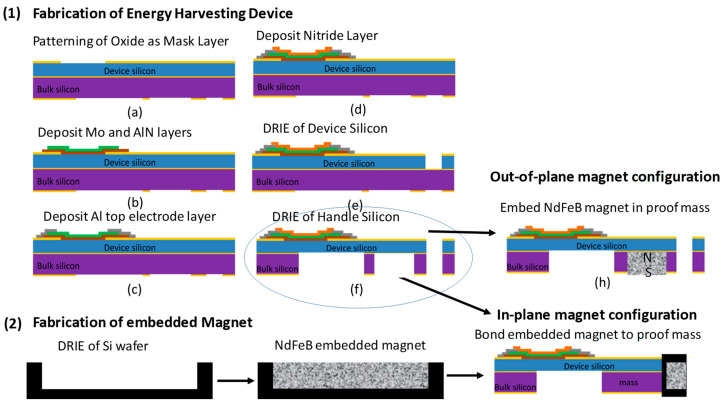
Fabrication of a (**1**) microelectromechanical system (MEMS) piezoelectric cantilever with embedded NdFeB magnets. Out-of-plane fabrication consisted of etching the cavity into the Si mass and embedding NdFeB powder capped with parylene. In-plane fabrication consisted of bonding a (**2**) silicon die with an embedded magnet on the end of the cantilever. AlN was used as the piezoelectric film.

The MEMS cantilever devices were fabricated using 100 silicon-on-insulator wafers with a 38-μm thick Si device layer, a 1 μm buried oxide layer, and a 525-μm thick Si handle layer. The fabrication process is shown in Figure 2, and was previously described in more detail [7,34,35]. A thermal oxide film was grown on the device and handle silicon to act as a mask layer during deep reactive ion etch (DRIE) process. The thermal oxide was patterned to define the beam and mass dimensions. Then, Mo (100 nm) [36] and AlN (1 μm) [37] layers were deposited using a sputtering process, without breaking the vacuum. The AlN layer demonstrated a highly crystalline c-axis textured (002) orientation with an omega XRD scan full-width half-maximum of 1.6° and a piezoelectric coefficient (d_33,f_) of 5.3 pm·V^−1^, which was performed on a PM 300 Piezotest meter. The AlN layer was patterned using a combination of wet etching tetramethylammonium hydroxide (TMAH) and dry reactive ion etching. A top metal Al layer was deposited and patterned using DC sputtering. The Al layer acted as the top electrode of the piezoelectric capacitor, as well as the bond pad conducting layer. A compressive silicon nitride film was deposited using plasma enhanced chemical vapor deposition (PECVD) to act as a stress control layer to prevent buckling of the cantilever device. The DRIE of the device silicon was etched down to the buried oxide film, which defined the shape of the cantilever. The handle silicon was also etched using DRIE, which was used to form the mass and release the structure. In the case of the out-of-plane configuration, an open cavity in the mass was etched, which was later filled with magnetic film to create a thick magnetic layer. The overall device was 4.8 mm in width by 9.2 mm in length, and had a beam length of 3 mm. The handle silicon acted as the proof mass, and in the case of the out-of-plane configuration the proof mass consisted of the magnetic film.

The magnets used in this study were created using NdFeB powder (Magnequench Inc., Sinapore). The magnetic powder was deposited into a silicon cavity and capped with a 5-μm thick parylene N capping layer based on previously described methods [30,38]. Integration of the magnet into the cantilever device for the out-of-plane configuration was created by etching the cavity during the handle silicon etching process. The proof mass for the in-plane configuration was fabricated separately using a 525 μm silicon wafer with a thermal oxide layer on top. DRIE was used to create the cavity by etching 425 μm into the silicon substrate. The cavity was then filled with magnetic powder and a parylene capping layer. A magnetic die measuring 4 mm in width by 550 μm in length was diced and bonded to the end of the cantilever, as shown in Figure 2. Stationary substrates were developed using the magnetic die, with varying lengths of up to 5 mm. The magnetic dies were magnetized using an ASC Impulse Magnetizer, ASC Scientific, RI, USA with a 50 kgauss field. The magnets had a remanence value of ~0.7 T. The polarity of the magnets was controlled based on their orientation in the magnetizer.

### 2.3. Experimental Characterization

The energy harvester cantilever devices were diced and individually filled or bonded with magnetic samples with varying magnetic configurations, as demonstrated in Figure 1. After the devices were magnetized, they were bonded onto a custom-made PCB using room temperature epoxy in order to avoid exceeding the Curie temperature of the magnet. The devices were then wire bonded to form an electrical interconnect.

The energy harvester was attached to a vibration shaker (LW 139, Labworks Inc., CA, USA) with an integrated accelerometer for feedback control. The shaker controlled the frequency and acceleration that was applied. In all experiments, the acceleration was kept below 0.15 g in order to determine the effects at low acceleration. The AC voltage was monitored through an oscilloscope with a variable load resistor, with the impedance value matching the impedance of the cantilever to maximize power. Power was calculated from the root mean square (RMS) voltage and load resistance. The bandwidth of the varying energy harvester devices was measured by fitting a Gaussian curve to the experimental measured power as a function of frequency curves. The bandwidth was calculated based on the full-width half-maximum (FWHM) value of the power vs. frequency curves.

**Figure 3:**
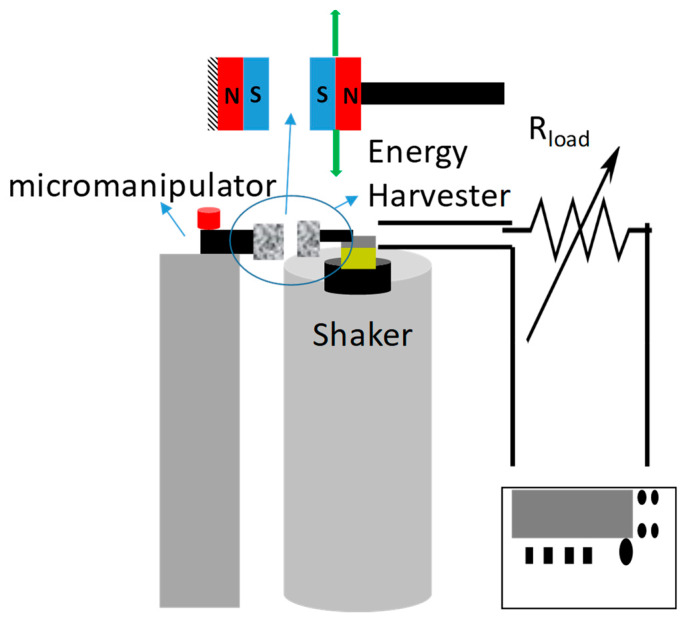
Schematic of the testing setup with a vibration shaker and energy harvester connected to a variable load resistor and oscilloscope. The tethered magnetic sample is connected to a micromanipulator to control spacing between magnets.

The schematic of the characterization setup is shown in Figure 3. The stationary magnetic samples were connected to a micromanipulator, which was used to vary the distance between the magnetic sample and the energy harvester. Calipers were used to measure the distance between the magnets. Alignment of the magnets was performed using a laser to ensure the devices were level. No significant buckling was visually noticeable.

## 3. Results and Discussions

**Figure 4:**
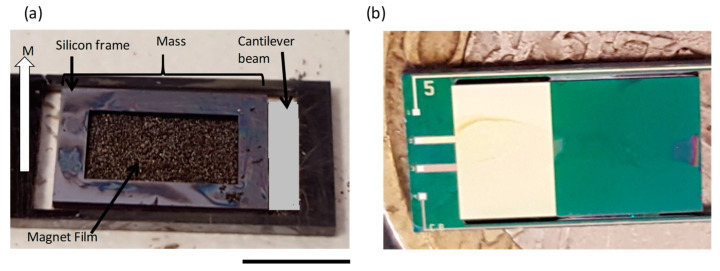
Image of fabricated device: (**a**) backside of the out-of-plane device with embedded magnetic material; (**b**) top view of the device with piezoelectric beam and large silicon mass. Scale bar is 5 mm.

The fabricated piezoMEMS cantilever energy harvesting devices are shown in Figure 4b, the piezoelectric capacitor beam is shown as the metallic area, while the other section is the large Si proof mass. Figure 4a demonstrates the backside magnetic filled cavity with a Si frame used in the out-of-plane configuration. The magnets were magnetized with polarity along the direction demonstrated in Figure 4a.

### 3.1. Nonlinear Simulation

**Figure 5:**
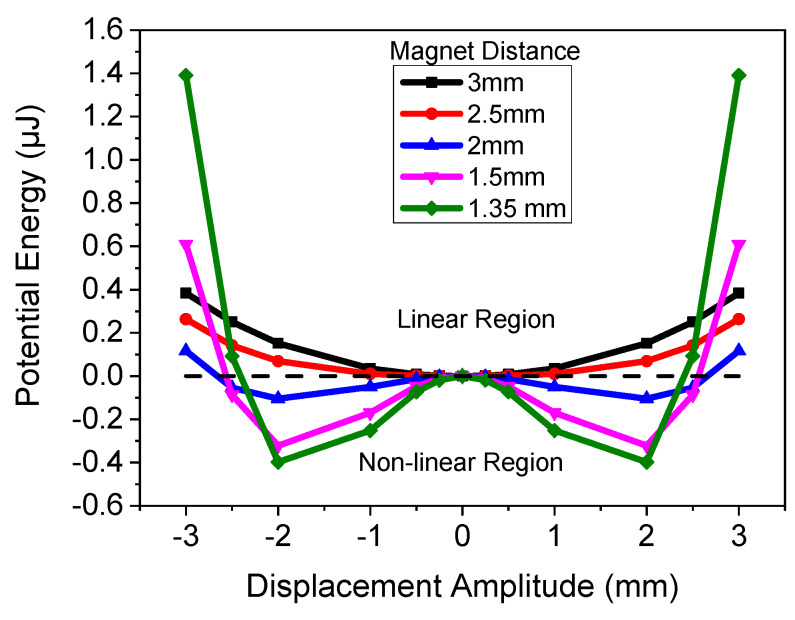
Numerical model of potential as a function of magnet spacing.

To determine the optimal magnetic spacing of the proof mass and the stationary magnets, the potential energy as a function of cantilever displacement was numerically estimated based on the following formula from [39,40]:

(1) $$U\left(y\right)=\frac{1}{2}\left(k-\frac{F_{M}}{\delta }\right)y^{2}+\frac{1}{4}\frac{F_{M}}{{2\delta }^{3}}y^{4}$$

where *U*(*y*) is the potential energy, *F_M_* is the magnetic force, *δ* is the distance between magnets, *y* is the displacement of the cantilever, and *k* is the stiffness of the rectangular beam, given by:

(2) $$k=\frac{Ewt^{3}}{4L^{3}}$$

where *w*, *t*, and *L* are the width, thickness, and length of the cantilever, respectively; and *E* is the elastic modulus of silicon, which was taken to be 160 GPa. The Si elastic modulus was used for the cantilever, as the Si thickness was much greater than that of the thin film AlN capacitor. The width, length. and thickness were 4.8 × 9.2 × 0.038 mm, respectively. Figure 5 demonstrates the results of the simulated equation for the in-plane single stationary mass, with varying magnetic spacing. The results show the potential spacing requirements needed to create a nonlinear vibrating system based on the embedded magnets used in this application. The results demonstrate that a magnet distance of >2.5 mm results in a linear vibrating system due to the weak magnetic force, whereas when the magnets are placed at a distance of <2.5 mm, the cantilever can be modeled as a nonlinear system with two potential wells. This demonstrates that as the magnets get closer together, the bandwidth should increase due to the nonlinearity. The simulation also demonstrates that as the cantilever amplitude increases to >2.5 mm, the potential will be in the linear region, as the magnetic field between the two magnets will decrease to a value that does not significantly impact the system. If commercial magnets with higher remanence values were used, the spacing could be increased as the magnetic force would be larger.

### 3.2. In-Plane Single Tether Magnet Configuration

**Figure 6:**
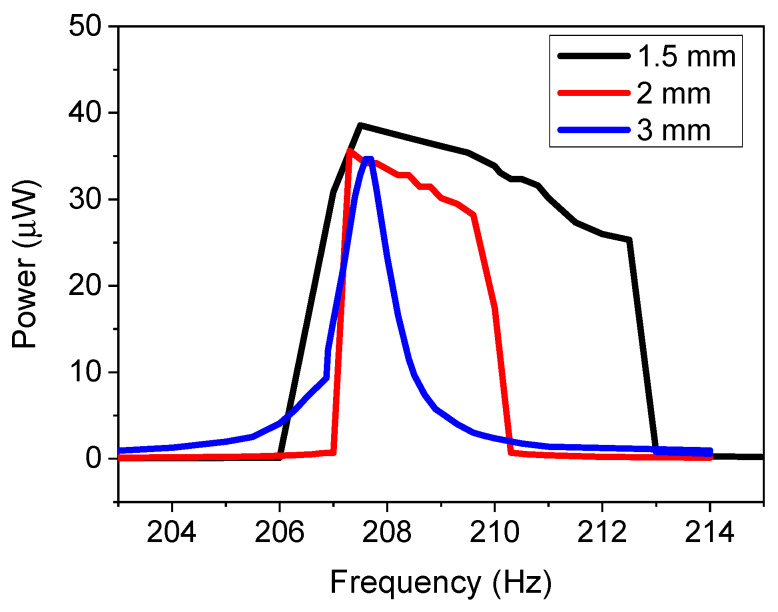
Experimental results of power for the in-plane single-tether configuration as a function of frequency for varying magnet spacing distances at an acceleration of 0.1 g.

To validate the model, the in-plane single stationary configuration with repulsive force magnets was experimentally investigated at an acceleration of 0.1 g. The displacement amplitude of the cantilever was approximately 1.5 mm, which was measured using scale bars and a high-speed camera. Three different magnetic spacings were investigated (1.5, 2, and 3 mm). The results for the power as a function of frequency are demonstrated in Figure 6. The results demonstrate that the 3 mm spacing resulted in a linear system with a FWHM value of 1.14 Hz, as was predicted by the model. This was because the magnetic force between the magnets was not strong enough for this spacing. As the magnetic spacing decreases, a nonlinear system was demonstrated for both 2 and 1.5 mm spacing. The bandwidth increased from 1.14 Hz for the linear system to 3.5 and 7.5 Hz for the 2 and 1.5 mm spacing, respectively. The results validate the simulated model. The different spacings resulted in a power density of 1.55, 1.43, and 1.39 μW·mm^−3^ for the 1.5, 2, and 3 mm spaced magnets at 0.1 g acceleration.

**Figure 7:**
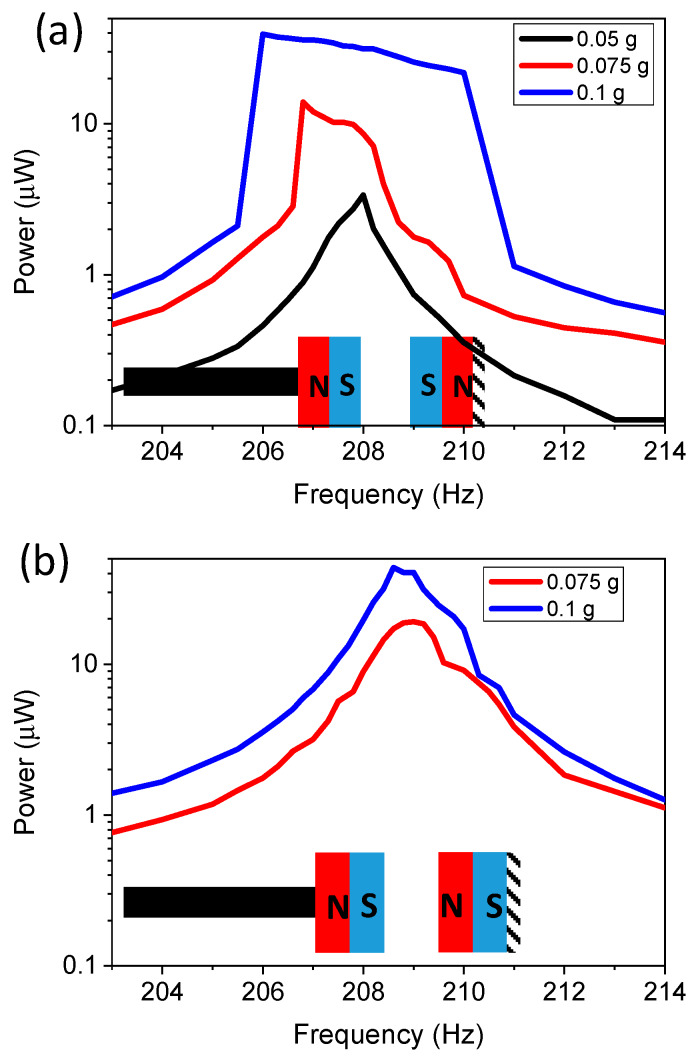
Experimental results of power as a function of frequency for an in-plane single-tether configuration for: (**a**) a repulsive magnetic configuration with varying acceleration; and (**b**) an attractive magnet configuration with varying acceleration.

To determine the effects of varying acceleration and varying magnetic poling configurations, the in-plane single stationary mass was implemented. The results are demonstrated in Figure 7. Figure 7a demonstrates the results for the repulsive magnetic configuration. A magnetic spacing of 1.75 mm was kept constant throughout the experiment. Acceleration was varied from 0.05 to 0.1 g. Acceleration values of >0.15 g caused the device to behave similarly to a linear cantilever, as the tip displacement was >2.5 mm. The results demonstrated that increased acceleration caused a wider bandwidth, with values of 1.7, 2.9, and 5.6 Hz for 0.05, 0.075, and 0.1 g, respectively. This demonstrates that in order to widen the bandwidth, a threshold acceleration was required, as the 0.05 g acceleration bandwidth gave similar bandwidths as the linear device. However, too much acceleration also caused similar linear effects, as the magnetic force no longer had a significant impact if the displacement was too large. The repulsive forces increase the bandwidth, as they create a force that acts on the cantilever, which is additional to the linear acceleration force caused from the applied vibration. Combining these forces results in a nonlinear dynamic structure. The repulsion force was highest at the original starting position (tip displacement = 0) in this configuration. As the magnetic proof mass increases the displacement amplitude, the magnetic force component was reduced due to increased spacing between the magnets. The exact nonlinear theory mechanism is a complex system that needs to be further explored in the future.

In order to investigate the effects of attractive magnetic forces on an in-plane configuration with a single stationary mass, the polarity of the magnet on the stationary sample was reversed. The acceleration was varied and the spacing was kept at 1.75 mm. The acceleration of 0.05 g is not demonstrated in Figure 7b, as the linear effects shown in Figure 7a demonstrate that the magnetic force does not influence the dynamics at that low of an acceleration, and the power results were the same as for the repulsive configuration. The results demonstrated in Figure 7b show that the bandwidth of the attractive magnetic force configuration was significantly decreased to 2.56 and 2.86 Hz for acceleration of 0.075 and 0.1 g compared to the repulsive magnetic force configuration (5.6 Hz at 0.1 g and 2.9 Hz at 0.075 g). However, the power density increased for the attractive force configuration from an average power density of 0.41 μW·mm^−3^ for the repulsive configuration at 0.075 g to a power density of 0.78 μW·mm^−3^ for the attractive configuration. A similar increase was also observed for 0.1 g acceleration, with results of 1.55 μW·mm^−3^ for the repulsive magnet and a power density of 1.63 μW·mm^−3^ for the attractive configuration. There was an increase in bandwidth of 2.86 Hz compared to the controlled sample of 1.14 Hz, which was believed to be due to the duffing resonator affect [9,13,41]. The attractive force from the magnets onto the cantilever created a stretching effect, which has previously resulted in increased bandwidth in energy harvesters. The increased power is believed to be due to amplitude control; as the displacement amplitude increased, the magnetic field force pulled the cantilever back towards the zero-displacement line or original starting point, so the cantilever had increased acceleration due to both magnetic force and vibrations. The results of widening the bandwidth by using repulsive magnetic forces matches well with previously reported data. However, macroscale devices have demonstrated wider bandwidths, which was due to using higher strength magnets and higher acceleration [24,39]. Previous reports of macroscale devices using both attractive and repulsive forces [42] in this configuration demonstrated similar effects of repulsive forces increasing the bandwidth and attractive forces generating narrower bandwidths. The critical distance required between magnets to result in nonlinearity was larger for the macroscale devices due to the magnet dimensions and remanence value of the magnets. Therefore, as demonstrated the MEMS-based device requires the magnets to be much closer together; increasing the magnet volume and strength would further improve this.

**Figure 8:**
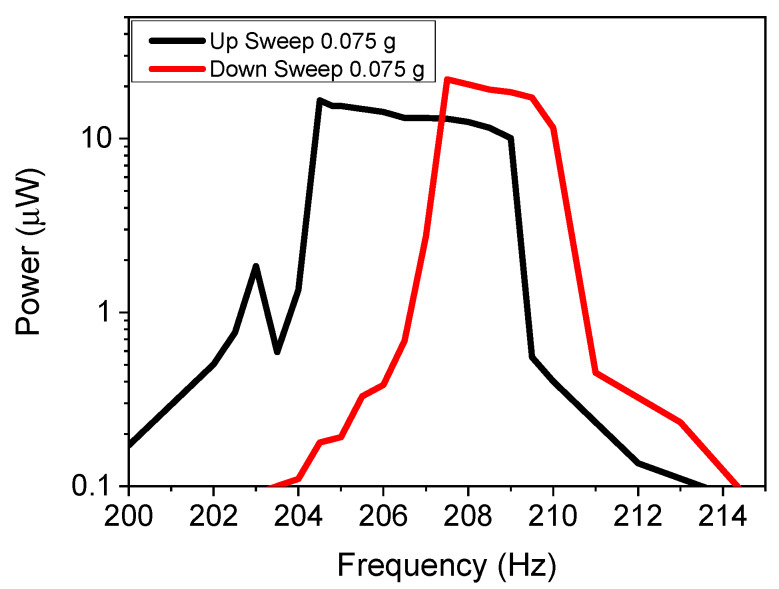
In-plane power as a function of frequency results for upwards and downwards sweeping frequencies for various acceleration values.

Nonlinear cantilevers demonstrated a hysteresis effect depending on frequency sweeping during the experimentation, so that if the cantilever had an applied increasing (upwards) frequency sweep vs. a decreasing (downward) sweep, the power vs. frequency curves varied. To validate that the system had a similar hysteresis effect, the cantilever with 1.5 mm spacing was swept from 200 to 214 Hz with a 0.01 Hz/s rate in both directions. The power vs. frequency curves are demonstrated in Figure 8. The results confirm that the upward frequency sweep differs from the downward sweep, with varying bandwidths of 5.2 and 4.4 Hz, respectively.

### 3.3. In-Plane Two-Tethered Magnet Configuration

**Figure 9:**
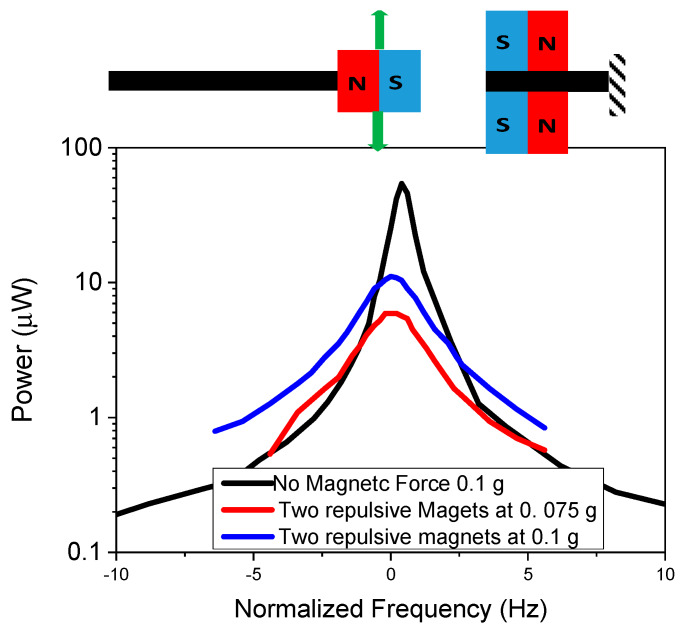
Experimental results of in-plane two-tethered configuration for repulsive magnets at varying acceleration.

The in-plane configuration with two stationary magnets was experimentally investigated to vary the acceleration of the repulsive magnetic force. A control with no magnetic stationary mass was used to compare the results with a traditional linear energy harvester. The results are demonstrated in Figure 9. The control (linear) energy harvesting cantilever had a FWHM of 0.94 Hz at 0.1 g, with a power density of 2.67 μW·mm^−3^. As expected, bandwidths increased with increasing acceleration, resulting in bandwidths of 3.5 and 2.5 Hz for 0.1 and 0.075 g acceleration. The bandwidth was lower compared to the single stationary magnetic mass configuration, especially for lower applied acceleration. This was because in this configuration the cantilever requires a significant tip displacement before the magnetic force can have a significant impact, so there was a threshold acceleration level that was needed to introduce the nonlinearity. The magnetic force at the starting tip location was lower, but as the cantilever displacement increased the magnetic force was increased as the spacing of the magnets was decreased up to a certain displacement. A closer magnet spacing could increase the bandwidth by increasing the magnetic force throughout the tip displacement range. In addition, curved or tapered stationary magnets could also further enhance performance.

### 3.4. Out-of-Plane Magnet Configuration

**Figure 10:**
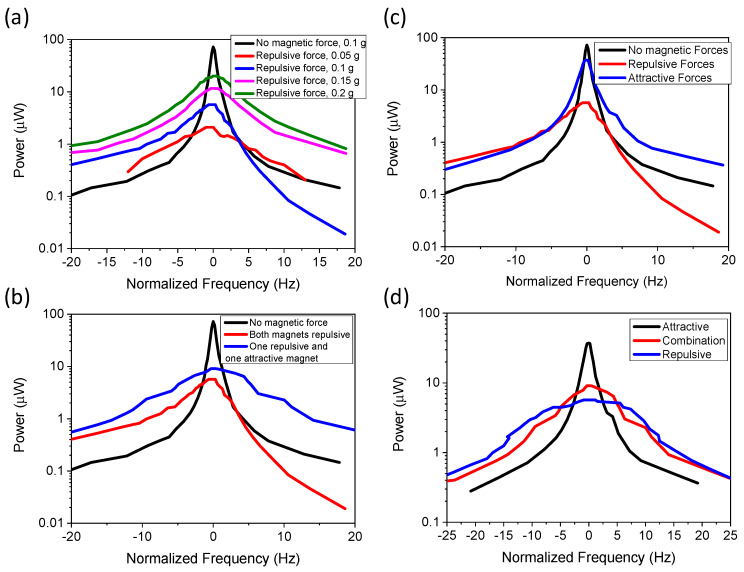
Experimental results for out-of-plane configuration: (**a**) for both repulsive forces with varying acceleration; (**b**) comparing repulsive forces with attractive and repulsive forces at 0.1 g; (**c**) comparing attractive forces and repulsive forces; (**d**) combining repulsive and attractive forces at 0.1 g.

The out-of-plane mode had an embedded magnetic mass and two stationary magnetic masses, which were spaced 2 mm away from the original starting point. The displacement amplitude did not exceed 1.5 mm in order to avoid the magnetic masses from contacting each other. This led to a decrease in power density, but the spacing could be altered to maximize the power density. The results of the various configurations are demonstrated in Figure 10. Figure 10a demonstrates the power as a function of normalized frequency with varying acceleration, where both stationary magnets generate a repulsive force on the cantilever. An energy harvester without a magnetic mass was used as the control, which gave a bandwidth of 0.94 Hz. Adding the repulsive magnetic forces caused a decrease in power, but the power values increased with increased acceleration, as was expected. The power decrease was expected, as the repulsive magnets reduced the displacement and acceleration of the device by repelling the acceleration force. The bandwidth was independent of the acceleration for this configuration, as it resulted in FWHM values of 6.2, 6.62, 6.4, and 6.2 for 0.05, 0.1, 0.15, and 0.2 g. This demonstrated that this configuration was ideal for higher acceleration, as the device can combine high power density with high bandwidth. The bandwidth increases as the repulsion forces generates a nonlinearity in the system, where the acceleration force and magnetic forces produce opposite effects.

Figure 10b compares the power and bandwidth for control magnets, repulsive magnets, and a combination of one repulsive and one attractive force at an acceleration of 0.1 g. The results demonstrate that for the combination of one repulsive and one attractive magnetic force resulted in higher power density with decreased bandwidth (4.88 Hz). However, the bandwidth was still significantly higher than the control (0.94 Hz). The power increased from 0.23 to 0.37 μW. Figure 10c demonstrates the configuration results for both repulsive and both attractive forces. The results demonstrate that the attractive force configuration had decreased bandwidth of 2.1 Hz compared to 6.62 Hz for the repulsive force configuration, but the power density increased from 0.23 to 1.36 μW·mm^−3^. The increased power was consistent with previous findings [33]. Power can be further increased by decreasing the magnetic spacing between magnets. This demonstrates that by creating combinations of attractive and repulsive forces, one can optimize the device to have high power or high bandwidth, or by using both forces the values can be balanced to enhance the power and bandwidth. 

Table 1 summarizes the results of this paper for devices under 0.1 g acceleration. The results demonstrate that repulsive magnetic forces caused an increase in bandwidth but a decrease in power density for all of the configurations. However, attractive forces demonstrated the opposite effect, with increased power density and decreased bandwidth. By combining magnetic forces, it is, therefore, possible to create a device that balances bandwidth and power density.

The control energy harvesting device developed in this manuscript had similar bandwidth as previous MEMS silicon-based piezoelectric devices, which had demonstrated bandwidths of about 0.5%–1% of the resonant frequency [4,5,34]. The power density of the control was also similar to previous reports of 2.5 μW·mm^−3^ [34] and 1.43 μW·mm^−3^ [4]. The in-plane configuration results demonstrated in this paper resulted in significantly higher power density compared to macroscale devices, with 0.168 [43], 0.41 [44], and 0.005 [45] μW·mm^−3^. However, the macroscale devices had higher FWHM bandwidths of ~5–10 Hz. This was believed to be due to the lower magnetic remanence value of the embedded magnets compared to sintered bulk NdFeB magnets, with remanence values >1.1 T. Therefore, there is a need to further develop higher remanence value magnets at the microscale. The out-of-plane configuration results are comparable to macroscale devices demonstrated in [25], which resulted in a power density of 0.173 μW·mm^−3^ and a bandwidth of 9 Hz. As to be expected, wider bandwidths come at the expense of decreasing the power density, so there are trade-offs that need to be balanced for specific applications. 

## 4. Conclusions

In conclusion, this paper demonstrated the ability to create a piezoMEMS energy harvesting device with embedded magnets that can be used in low acceleration (<0.1 g) applications. The paper demonstrated the effects on the bandwidth and power density with three different magnetic configurations. In addition, the paper demonstrated how varying the magnetic force from attractive to repulsive can affect the bandwidth and power for each of these configurations. The results of this paper demonstrate that attractive and repulsive forces can be used to optimize devices for specific power and bandwidth. Ideally, an energy harvesting device would have both high power density and bandwidth, but the two parameters are typically inversely linked. The fabrication process with embedded magnets demonstrated in this paper used multiple wafers to create the magnetic stationary samples, however future applications could investigate methods to combine these into a monolithic device.

## Figures and Tables

**Table 1:** Comparison of different configurations as demonstrated for 0.1 g. FWHM, full-width, half-maximum.

Device Configuration	Bandwidth (FWHM)	Power Density (μW·mm^−3^)
In-plane single (attractive)	2.86	1.63
In-plane single (repulsive) In-plane double (repulsive)	5.6 3.5	1.51 0.45
Out-of-plane (repulsive)	6.62	0.23
Out-of-plane (attractive)	2.1	1.36
Out-of-plane (combination)	4.88	0.37
Control	0.94	2.67
